# Breeding populations of Marbled Godwits and Willets have high annual survival and strong site fidelity to managed wetlands

**DOI:** 10.1002/ece3.9667

**Published:** 2023-01-18

**Authors:** Brett K. Sandercock, Cheri L. Gratto‐Trevor

**Affiliations:** ^1^ Department of Terrestrial Ecology Norwegian Institute for Nature Research Trondheim Norway; ^2^ Science and Technology Branch Environment and Climate Change Canada Saskatoon Saskatchewan Canada

**Keywords:** apparent survival, biometrics, demography, longevity, mark recapture, mate fidelity, shorebird, wader

## Abstract

The Prairie Pothole Region of central Canada supports a diverse community of breeding waterbirds, but many species have declining populations and the demographic mechanisms driving the declines remain unknown. We conducted a 7‐year field study during 1995–2001 to investigate the demographic performance of Marbled Godwits (*Limosa fedoa*) and Willets (*Tringa semipalmata*) breeding in managed wetlands near Brooks, Alberta. Mark‐recapture analyses based on Cormack–Jolly–Seber models revealed that the annual rates of apparent survival for Marbled Godwits ($\hat{\phi }$ = 0.953 ± 0.012SE) and Willets ($\hat{\phi }$ = 0.861 ± 0.015SE) are among the highest rates of survivorship reported for any breeding or nonbreeding population of large‐bodied shorebirds. Our estimates of life expectancy for males were comparable to longevity records in godwits (17.3 years ±5.8SE vs. 25–29+ years) and willets (7.7 ± 1.5SE vs. 10+ years). The two species both showed strong breeding site fidelity but differed in rates of mate fidelity. Pairs that reunited and males that switched mates usually nested <300 m from their previous nests, whereas females that switched mates usually moved longer distances >1.1–1.5 km. Returning pairs usually reunited in godwits (85%) but not in willets (28%), possibly because of species differences in adult survival or patterns of migration. Baseline estimates of annual survival for banded‐only birds will be useful for evaluating the potential effects of new tracking tags or the environmental changes that have occurred during the past 20 years. Conservation strategies for large‐bodied shorebirds should be focused on reduction of exposure to anthropogenic mortality because low rates of natural mortality suggest that losses to collisions at breeding sites or harvest at nonbreeding areas are likely to cause additive mortality.

## Graphical abstract

Demographic causes of population declines of wetland birds are poorly understood. We conducted a population study of Marbled Godwits and Willets breeding in southern Alberta. Annual survival rates were among the highest rates reported for any species of large‐bodied shorebird. Site and mate fidelity were high for birds breeding in managed wetlands. Conservation plans for shorebirds should prioritize reducing exposure to anthropogenic losses because losses to collision or harvest are likely to cause additive mortality.

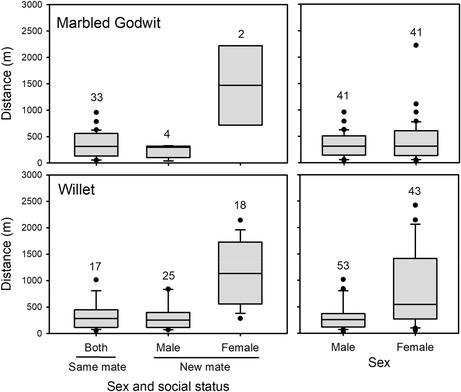

## 1 INTRODUCTION

The Prairie Pothole Region of central Canada is a complex landscape of semipermanent wetlands that provide important breeding habitat for migratory waterbirds, including high densities of nesting waterfowl, shorebirds, wading birds, and marsh songbirds. Habitat availability has been reduced by losses to drainage and conversion to cultivation or pasture (Doherty et al., 2018; Gratto‐Trevor, 2006), although conservation easements for waterfowl production have the potential to benefit a diversity of wildlife species (Koper & Schmiegelow, 2006; Tori et al., 2002). Habitat conditions in the Prairie Pothole Region are usually determined by annual variation in precipitation which affects soil moisture, water depth, and vegetative cover, but with predictions of drier conditions under future climate change (Niemuth et al., 2010). The local abundance and distribution of migratory waterbirds varies with annual conditions but many species are also showing long‐term population declines (Forcey et al., 2007; Niemuth et al., 2008; Steen et al., 2014). Major threats to migratory waterbirds are thought to include habitat loss and degradation, climate change, and unsustainable harvest (Bellio et al., 2017; Pearce‐Higgins et al., 2017; Watts et al., 2015), but the demographic mechanisms of ongoing population declines remain poorly understood. Reliable estimates of fecundity and survival are fundamental to conservation planning but remain unavailable for many widespread species (Méndez et al., 2018; Piersma et al., 1997; Sandercock, 2003). Baseline information on demographic rates for stable populations are particularly useful for identifying environmental perturbations that are likely to reduce future population viability (Burton et al., 2006; McDuffie et al., 2022; Piersma et al., 2016; Taylor & Dodd, 2013).

**FIGURE 1:**
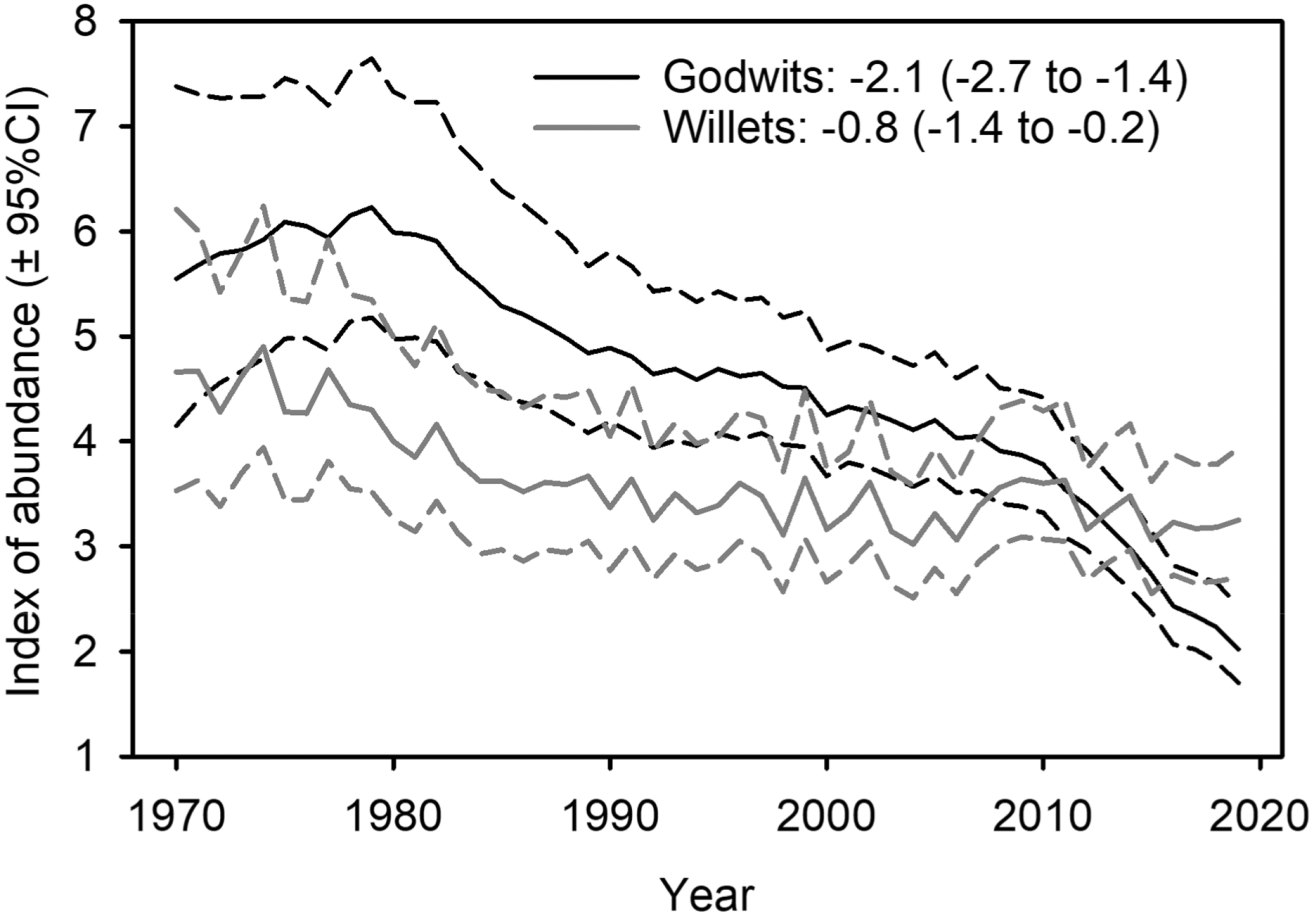
Population indexes and long‐term trends from the Breeding Bird Survey program for Marbled Godwits and Willets in the Prairie Pothole Region of Canada (BCR 11), 1970–2019. *Source*: Smith et al. (2020).

Marbled Godwits (*Limosa fedoa*) and Willets (*Tringa semipalmata*) are two species of large‐bodied shorebirds that breed in the Prairie Pothole Region of central Canada and the USA (Gratto‐Trevor, 2006; Specht et al., 2020). The biogeography of these species in North America is complex with three separate breeding populations of Marbled Godwits and two disjunct breeding populations of Willets (Gibson & Kessel, 1989; Martínez‐Curci et al., 2014; Oswald et al., 2016). The Prairie Pothole Region supports large breeding populations of the midcontinental subspecies (*L. f. fedoa* and *T. s. inornata*), and Canada has significant responsibility for stewardship of these birds. Breeding populations of godwits and willets have been declining at −0.8% to −2.1% per year in the Prairie Pothole Region of Canada during the last half century (Figure 1), although nonbreeding populations in coastal California appear to be more stable (Warnock et al., 2021). Both species are categorized as species of ‘High Concern’ in assessments of Shorebirds of Conservation Concern in Canada (Hope et al., 2019) and the United States (US Shorebird Conservation Plan, 2016).

Large‐bodied shorebirds are often characterized by a “slow” life‐history strategy with low reproductive rates, delayed maturity, and high survivorship (Myers et al., 1987; Watts et al., 2015). Shorebirds with socially monogamous mating systems also tend to have strong fidelity to breeding sites and to mates (Kwon et al., 2022; Oring & Lank, 1984). Thus, population growth rates are likely to be more sensitive to factors that affect adult survival and breeding site fidelity than components of reproduction (Hitchcock & Gratto‐Trevor, 1997; Koivula et al., 2008; Ottvall & Härdling, 2005). Previous population studies of Marbled Godwits and Willets have been limited but have provided basic information on migratory connectivity (Gratto‐Trevor, 2011; Haig et al., 2002; Olson et al., 2014), habitat preferences (Gratto‐Trevor, 2006; Ryan & Renken, 1987; Specht et al., 2020), nesting success (Garvey et al., 2013; Kantrud & Higgins, 1992), and return rates or longevity (Colwell et al., 1995; Howe, 1982; Kelly & Cogswell, 1979). No previous analyses of adult demography exist for the prairie populations of Marbled Godwits or Willets. The three objectives of our field project were (i) to evaluate use of biometrics for sexing of adult birds, (ii) to conduct mark‐recapture analyses to estimate apparent survival corrected for variation in the probability of encounter, and (iii) to use records of returning birds to compare patterns of mate and site fidelity between the two species.

## 2 METHODS

### 2.1 Study area and annual conditions

We conducted a 7‐year breeding study (1995–2001) of Marbled Godwits and Western Willets at the Kitsim wetland complex, located 13 km southwest of Brooks, Alberta (50.5042°N, 112.0459°W). The core study area was northwest of the intersection of highways 539 and 36 and had an area of 21.6 km^2^ (3.3 km W–E × 6.5 km N–S). Additional monitoring was conducted on two smaller patches to the west (2.7 km^2^) and to the southeast (4.4 km^2^) of the core study area. The semi‐arid habitats included native mixed‐grass prairie interspersed with a network of ca. 50 small wetlands ranging in size from 16–120 ha. The study site was managed by Ducks Unlimited Canada for breeding waterfowl and cattle production, with flooding in spring and autumn via irrigation canals connected to the Bow River. Other infrastructure included pumpjacks and storage tanks for oil and gas extraction and transmission powerlines.

Annual variation in wetland conditions and small mammal numbers were assessed at a regional level (Table 1). For an index of wetland conditions, we used pond counts recorded in May for stratum 28 (ca. 40 K km^2^) in the annual Waterfowl Breeding Population and Habitat Surveys coordinated by the Canadian Wildlife Service. High numbers of microtine rodents were observed during field work in 1997 but were low in other years. For an index of rodent activity and predator numbers, we compiled information on the relative abundance of Short‐eared Owls (*Asio flammeus*) from June surveys in Alberta conducted under the Breeding Bird Survey program (Specht & Arnold, 2018). Potential nest predators observed in the area included several species of gulls (*Larus* spp.), Richardson's ground squirrels (*Urocitellus richardsonii*) and coyotes (*Canis latrans*), whereas predators of adults included Short‐eared Owls, Northern Harriers (*Circus hudsonicus*) and Swainson's Hawks (*Buteo swainsoni*). Field work at Kitsim was conducted by 2–6 shorebird observers from late April to mid July with 37–67 days of nest searching, monitoring and captures in 1995–2000, and 18 days of resighting effort in 2001.

**TABLE 1:** Annual variation in wetland conditions, predator abundance, and field effort during a 7‐year field study near Brooks, Alberta, 1995–2001.

Year	No. of ponds in May (1000s)	Rodent numbers	Abundance of Short‐eared Owls	No. days afield	Seasonal period of field work
1995	58.8	Low	0.02 (0.00, 0.08)	37	4 May–30 Jun
1996	130.5	Low	0.08 (0.03, 0.19)	57	3 May–19 Jul
1997	143.2	High	0.66 (0.25, 2.19)	57	2 May–11 Jul
1998	66.3	Low	0.03 (0.01, 0.09)	58	22 Apr–14 Jul
1999	67.2	Low	0.08 (0.03, 0.29)	63	20 Apr–16 Jul
2000	63.1	Low	0.09 (0.04, 0.22)	67	18 Apr–14 Jul
2001	56.6	Low	0.02 (0.00, 0.08)	18	23 Apr–6 Jul

### 2.2 Field methods

The study site at Kitsim was searched for nests twice per year during the first 6 years of the study. Nests were found by cable dragging with a 30 m cable or chain dragged slowly between a pair of all‐terrain vehicles. At first discovery, nest sites of shorebirds and waterfowl were marked with a pin flag. Nest locations were recorded on high‐resolution aerial photos and later digitized with UTM coordinates for UTM zone 12 in eastern Alberta (±0.1 m). All adult godwits and about half of the adult willets were captured at nest sites during early incubation by lowering a mist net over the nest. The remaining willets were captured at their nests with passive walk‐in traps made from chicken wire. At first capture, each adult was individually marked with a unique combination of three color bands, a white flag with two bands on each tibia, and a numbered stainless‐steel band on the tarsus. The wrap‐around color rings were not sealed, but only one Willet lost a color ring during the course of the study. Willets were also temporarily marked with pink or yellow dyes on their white wing bars or rump for easier identification in flight and to reduce unnecessary disturbance from re‐trapping. After capturing one parent, nests were revisited to capture any unbanded mates. In the case of godwits, females tended to incubate during the day and males at night so it was possible to capture mates by visiting the nest at different times of day. In the case of Willets, we attempted to trap the mate if a flushed bird did not have a temporary plumage mark. No nests were deserted because of nest monitoring or capture of adults at the nest.

In the last six years of the project (1996–2001), we conducted regular surveys during the prelaying and brood attendance periods to search for returning adults that had been banded in previous years. Godwits and willets encountered at foraging sites, nests, or attending broods were routinely scanned for color bands. We recorded color‐band combinations, locations, and behavior for all banded individuals. Mated pairs were identified during the laying period by mate‐guarding behavior and during the incubation and the brood‐rearing periods by joint parental care. In addition, the larger wetland complexes surrounding the core study area were surveyed three times per year for banded birds by driving systematic survey routes with all‐terrain vehicles (Gratto‐Trevor, 2006).

### 2.3 Morphometrics

Godwits and willets were sexed by morphometrics recorded in the field, including body mass, culmen (feathering to tip), maximum wing chord (flattened and straightened), tarsus, and vent length. Preliminary sex assignments were confirmed in the field by behavioral observations of aerial displays and wing flashing by males, sex roles of males and females during mate guarding and copulations, and by comparing the morphometrics of birds in mated pairs and their partners in different years. Sexing with morphometrics was also validated with genetic methods for a subset of birds captured in Alberta and with related work in other populations (Ayala‐Pérez et al., 2013; Haig et al., 2002). We estimated relative differences between the sexes with the esc_mean_sd function in the esc package of Program R, where the units of effect size for Hedges' *g* are in standard deviations.

### 2.4 Mark‐recapture analyses

Resightings of birds during the breeding season were combined with recaptures of birds during incubation to create live encounter histories for each individual bird. Each year within the encounter history was coded as 1 = resighted or captured on the study area, or 0 = not detected during the breeding season. All birds included in the encounter histories were first captured and banded as adults of unknown age (after‐hatch‐year or AHY). Birds marked as flightless young were not included in the survival analysis because no godwits and only two willets were encountered again in the study area. In the case of known mortality events where an adult bird was found dead during the breeding season, we censored the individual as not released after the occasion with the dead recovery event. We used Cormack–Jolly–Seber (CJS) models to estimate annual probabilities of apparent survival (*ϕ*), corrected for the probability of encounter (*p*, Sandercock, 2020). Apparent survival is the joint probability of true survival (*S*) and site fidelity (*F*) to the study area between two consecutive years. The complement of apparent survival (1−*ϕ*) included losses to mortality and to permanent emigration caused by dispersal from the study site. The probability of encounter is the product of site propensity and the true detection rate, and the complement (1−*p*) included losses due to temporary emigration or incomplete detection.

Mark‐recapture analyses were conducted in Program Mark (ver. 9.0) in an information theory framework (White & Burnham, 1999). First, we selected factors to include in the global model for the probabilities of apparent survival (*ϕ*) and encounter (*p*). We modeled both parameters as a function of sex (sex) because males and females were expected to differ in sex roles during the breeding season and in timing of migration and use of wintering areas during the nonbreeding season. Males may have higher apparent survival and detection rates than females because the sexes differ in reproductive effort, males tend to have stronger site fidelity in male‐territorial species, and males are more active in display, mate defense, and brood attendance (Sandercock et al., 2000). We did not consider time‐since‐marking models for apparent survival because few birds had encounter histories where they were detected on only a single occasion (godwits: 4 of 94, 4.3%; willets: 30 of 175, 17.1%). We modeled *ϕ* and *p* with time‐dependence (year) to test for patterns of annual variation. Further, we modeled apparent survival as a function of number of ponds and Short‐eared Owls as annual indices of wetland conditions and predator abundance (Table 1). Number of ponds was bimodal so we grouped years into two bins and compared apparent survival between five years with typical water levels (1995 and 1998–2000: 56.6–67.2 K ponds) vs. two years with particularly wet conditions (1996–1997: 130.5–143.2 K ponds). Similarly, numbers of rodents and Short‐eared Owls were high in only one year so we grouped years into two bins and compared apparent survival between the rodent peak (1997) vs. all other years (1995–1996 and 1998–2000). Last, we modeled encounter rates as a function of the number of days afield as an index of field effort (Table 1, 1996–2001: 18–67 days afield). We combined factors in main effect models (+) and factorial models with interaction terms (×).

Our global model was the standard CJS model with the effects of sex and year in both parameters: *ϕ*(sex × year), *p*(sex × year). We used Program Release to evaluate the goodness‐of‐fit of the global model to the encounter histories, and calculated an overdispersion factor as the ratio of the component tests to their degrees of freedom ($\hat{c}=\sum \chi^{2}/\sum \mathrm{df}$). Moderate amounts of overdispersion are common in mark‐recapture data and values of $\hat{c}$ < 3 indicate that the global model is acceptable. The asymptotic value of $\hat{c}$ is expected to be one in the case of no overdispersion (Sandercock, 2020).

We proceeded with model testing by fitting reduced models with fewer parameters. All models were constructed with design matrices and the logit‐link function. Starting with our global model, we applied constraints to the probabilities in the following order: encounter rates (*p*) and then apparent survival (*ϕ*). Each probability was modeled by dropping interaction terms from factorial models (sex × year) to create additive models (sex + year) and then by dropping main effects to create single factor (sex or year) or constant models (con). Once we had identified a minimum AICc model, we added factors back in parameters to explore whether nearby models were a better fit. Parameter counts (*K*) were adjusted to match the structure of the model which was usually the number of columns in the design matrix. In the special case of models with annual variation in both apparent survival and the probability of encounter, we also adjusted *K* to account for inestimable parameters from the last transition of the CJS models (Sandercock, 2020). The parameter count (*K*) and the log‐likelihood ‐2ln(*L*) were combined to calculate Akaike's Information Criterion (AICc) and models were ranked by the difference from the minimum AICc model (ΔQAICc). Akaike weights (*w*_*i*_) were used to determine the relative likelihood of a model within the set of candidate models, and ratios of weights between groups of candidate models with or without explanatory factors ($\sum w_{j}/\sum w_{i}$) were used to quantify the relative support for different effects.

To examine patterns of variation between the sexes and annual conditions, we took estimates of apparent survival ($\hat{\phi }$) and encounter rates ($\hat{p}$) from factorial models such as *ϕ*(sex × ponds), *p*(con), even if the unconstrained model was a relatively poor fit. Models were considered equally parsimonious if ΔAICc ≤ 2, but we also examined the coefficients for the explanatory variable in cases where models differed by a single parameter (Δ*K* = 1, Arnold, 2010). To obtain overall parameter estimates, we reran the best‐fit models with the MCMC sampling procedure of Program Mark and present estimates as means ±1SE with 95% credible intervals (95%CrI). We fitted MCMC models with a single chain and 40,000 tuning samples, 10,000 burn‐in samples and 100,000 stored samples for the posterior distributions. We also used the variance components procedure of Program Mark to estimate process variance of the overall estimates of apparent survival. Here, the method of moments approach was applied to the time‐dependent estimates of apparent survival from model *ϕ*(year), *p*(con). We used the analysis of deviance procedure in Program Mark to estimate the percentage of annual variation in apparent survival that was explained by the annual covariates for number of ponds and relative abundance of owls. The ANODEV procedure was used to compare three nested models, *ϕ*(year), *ϕ*(covar), and *ϕ*(con), and the calculations were based on differences in deviances and degrees of freedom. Life expectancy of adults ($\hat{E}$) and 95% credible intervals were calculated as derived parameters from the mean estimates of apparent survival ($\hat{\phi }$) with 2.5% and 97.5% quartiles from the posterior distributions, where the SE($\hat{E}$) was calculated with a function based on the delta method (Powell, 2007):

$$\hat{E}=\frac{-1}{\ln\left(\hat{\phi }\right)}\;\mathrm{SE}\left(\hat{E}\right)=\sqrt{{\left(\mathrm{SE}\left(\hat{\phi }\right)\right)}^{2}{\left(\frac{1}{\hat{\phi }{\left(\ln\left(\hat{\phi }\right)\right)}^{2}}\right)}^{2}}$$

### 2.5 Mate fidelity and site fidelity

To determine patterns of mate fidelity and breeding site, we examined mating histories for pairs of marked birds where at least one partner returned to the study site in the same or a following year. Pairs were considered to have reunited with the *same mate* if both members of the pair returned and were recorded together on a nest. Males and females were considered to have changed partners if at least one bird returned and mated with a *new mate*. Mate change may have been caused by absence of a mate that died or to divorce if both partners returned but did not reunite. If only one parent was captured or observed at a nest, the pair status was considered *unknown*. In a few cases, pairs had a mating history with a nest where only one parent was detected but we considered pairs to have stayed together if they were recorded as a pair at a prior and later nest. Breeding site fidelity was calculated from UTM coordinates as the linear distance between nesting attempts in different years. We first compared breeding dispersal distances by social status for reuniting pairs, males that changed mates and females that changed mates. We then combined the three classes with birds of unknown social status to compare patterns of breeding site fidelity between the sexes. Analyses of breeding site fidelity were conducted using the base functions of Program R (ver. 4.2.0, R Core Team, 2022), including functions for t‐tests with unequal variance (t.test) and one‐way analysis of variance (aov).

## 3 RESULTS

### 3.1 Morphometrics

Godwits were dimorphic in body size and coloration (F/M = 1.06–1.29), and the best character traits for distinguishing between the sexes were culmen length (Hedge's *g* = 4.32) and body mass (*g* = 2.55, Table 2). Females had longer bills with dark coloration (range = 108.9–130.9 mm, *n* = 49), whereas males had shorter bills with more orange at the base (89.3–108.5 mm, *n* = 46). The sexes overlapped in body mass, but females were 19% heavier than males on average. In contrast, Willets had greater overlap in body size (F/M = 1.02–1.29) and no sexual differences in coloration. The best characters for distinguishing the sexes in Willets were body mass (Hedge's *g* = 1.76) and length of the vent after egg laying (*g* = 1.44). Females tended to be 11% heavier and had vents that were 34% longer than males.

**TABLE 2:** Morphometrics, sexual size dimorphism (F/M), and standardized mean effect size (Hedge's *g*) for adult Marbled Godwits and Willets captured at the Kitsim wetland complex near Brooks, Alberta, 1995–2000.

Variable	Females			Males			F/M	Hedges' *g* ± SE
	Mean	SD	*n*	Mean	SD	*n*		
*Marbled Godwits*								
Mass (g)	388.3	25.4	49	327.8	21.3	46	1.185	2.55 ± 0.28
Culmen (mm)	119.7	5.5	49	98.1	4.3	46	1.220	4.32 ± 0.38
Wing (mm)	252.8	5.9	48	239.4	5.5	46	1.056	2.33 ± 0.27
Tarsus (mm)	77.4	3.4	49	72.7	2.5	46	1.065	1.55 ± 0.23
Vent length (mm)	10.7	1.7	49	7.8	1.4	46	1.375	1.92 ± 0.25
*Willets*								
Mass (g)	285.7	17.8	86	257.9	13.1	80	1.108	1.76 ± 0.18
Culmen (mm)	61.6	2.4	86	60.4	2.1	80	1.020	0.53 ± 0.16
Wing (mm)	221.8	4.9	86	216.8	4.1	80	1.023	1.10 ± 0.17
Tarsus (mm)	67.2	2.5	86	65.9	7.2	80	1.019	0.24 ± 0.16
Vent length (mm)	11.7	2.4	86	8.7	1.5	80	1.340	1.44 ± 0.17

### 3.2 Return rates

A total of 94 Marbled Godwits and 175 Willets were captured and color banded at the Kitsim wetland complex during the first 6 years of the 7‐year study period. A majority of birds survived and returned to the study area at least once after the year of first capture with higher return rates among godwits (90 of 94, 95.7%) than willets (145 of 175, 82.9%, Fisher's Exact test: *p* = .002). Return rates tended to be higher among male (45 of 46, 97.8%) than female godwits (45 of 48, 93.8%, Fisher's exact test: *p* = .62), and also among male (70 of 84, 83.3%) than female willets (75 of 91, 77.8%, *p* > .99). Mortality events during the breeding season were recorded for a total of 14 banded birds, including three godwits (2F, 1 M) and 11 willets (5F, 6 M). In addition, five unbanded birds were found dead in the course of field work including two godwits (2F) and three willets (1 M, 2 U). Cause of death for the 19 mortality events included being killed by a predator (10, 52.6%), collisions with powerlines or possible scavenging of a collision mortality (8, 42.1%), and one godwit that was killed in a hail storm (1, 5.3%). Of the 10 birds killed by predators, nine were found dead near their nests including a direct observation of a Willet that was depredated by a Swainson's Hawk.

### 3.3 Mark‐recapture analyses

Preliminary GOF tests with the component tests of Program Release indicated that the standard CJS model *ϕ*(sex × year), *p*(sex × year) was a good fit to the encounter histories for both Marbled Godwits ($\chi_{20}^{2}$ = 8.59, *p* = .987) and Willets ($\chi_{23}^{2}$ = 20.2, *p* = .628). Estimates of the overdispersion factor based on the ratio of the *χ*‐statistic to the degrees of freedom were less than one, so we set $\hat{c}$ = 1 and used AICc for model selection.

Models with a constant probability of encounter were a better fit than models where the parameter was constrained as a function of sex, year or annual variation in field effort (ratio of AICc weights, godwits: 0.97/0.07 = 13.9×, willets: 0.81/0.19 = 4.3×, Table 3). Estimates of the probability of encounter were high for both species of shorebirds and were $\hat{p}$ = 0.919 ± 0.017SE in godwits and $\hat{p}$ = 0.887 ± 0.019SE in willets.

**TABLE 3:** Model selection for Cormack–Jolly–Seber models for estimation of apparent survival (*ϕ*) and probability of encounter (*p*) for adult Marbled Godwits and Willets at the Kitsim wetland complex near Brooks, Alberta, 1995–2001.

Model structure		Model parameters				
*ϕ*	*p*	*K*	−2ln(*L*)	AICc	ΔAICc	*w*_*i*_ ≤
*Marbled Godwits*						
con	con	2	327.4	331.5	0.0	0.258
sex	con	3	326.5	332.6	1.1	0.148
ponds	con	3	326.8	332.9	1.4	0.125
owls	con	3	327.0	333.1	1.6	0.116
sex	effort	4	325.6	333.7	2.2	0.085
sex + ponds	con	4	326.0	334.1	2.6	0.070
sex + owls	con	4	326.1	334.2	2.7	0.066
sex × owls	con	5	324.9	335.1	3.7	0.042
year	con	7	321.4	335.7	4.3	0.031
sex × ponds	con	5	325.6	335.8	4.3	0.029
sex + year	con	8	320.5	337.0	5.5	0.017
sex	year	8	321.1	337.6	6.1	0.012
sex × year	sex × year	22	305.5	352.7	21.2	0.000
*Willets*						
sex	con	3	622.7	628.8	0.0	0.229
sex + ponds	con	4	621.6	629.6	0.8	0.150
sex	effort	4	621.6	629.7	0.9	0.149
con	con	2	626.4	630.4	1.6	0.103
sex + owls	con	4	622.7	630.8	2.0	0.083
ponds	con	3	625.0	631.1	2.3	0.073
sex × ponds	con	5	621.5	631.7	2.9	0.054
owls	con	3	626.4	632.4	3.6	0.037
sex	year	8	616.2	632.5	3.7	0.036
sex × owls	con	5	622.5	632.6	3.8	0.034
sex + year	con	8	616.3	632.6	3.8	0.034
year	con	7	619.8	634.1	5.3	0.016
sex × year	sex × year	22	598.2	644.6	15.8	0.000

*Note*: Alternative models included the effects of group (sex), time (year), annual covariates (ponds, owls), and intercept‐only constant model (con). Models that received little support are not shown (ΔAICc > 10 and *w*_*i*_ < 0.005), except for the global starting model *ϕ*(sex × year), *p*(sex × year).

**FIGURE 2:**
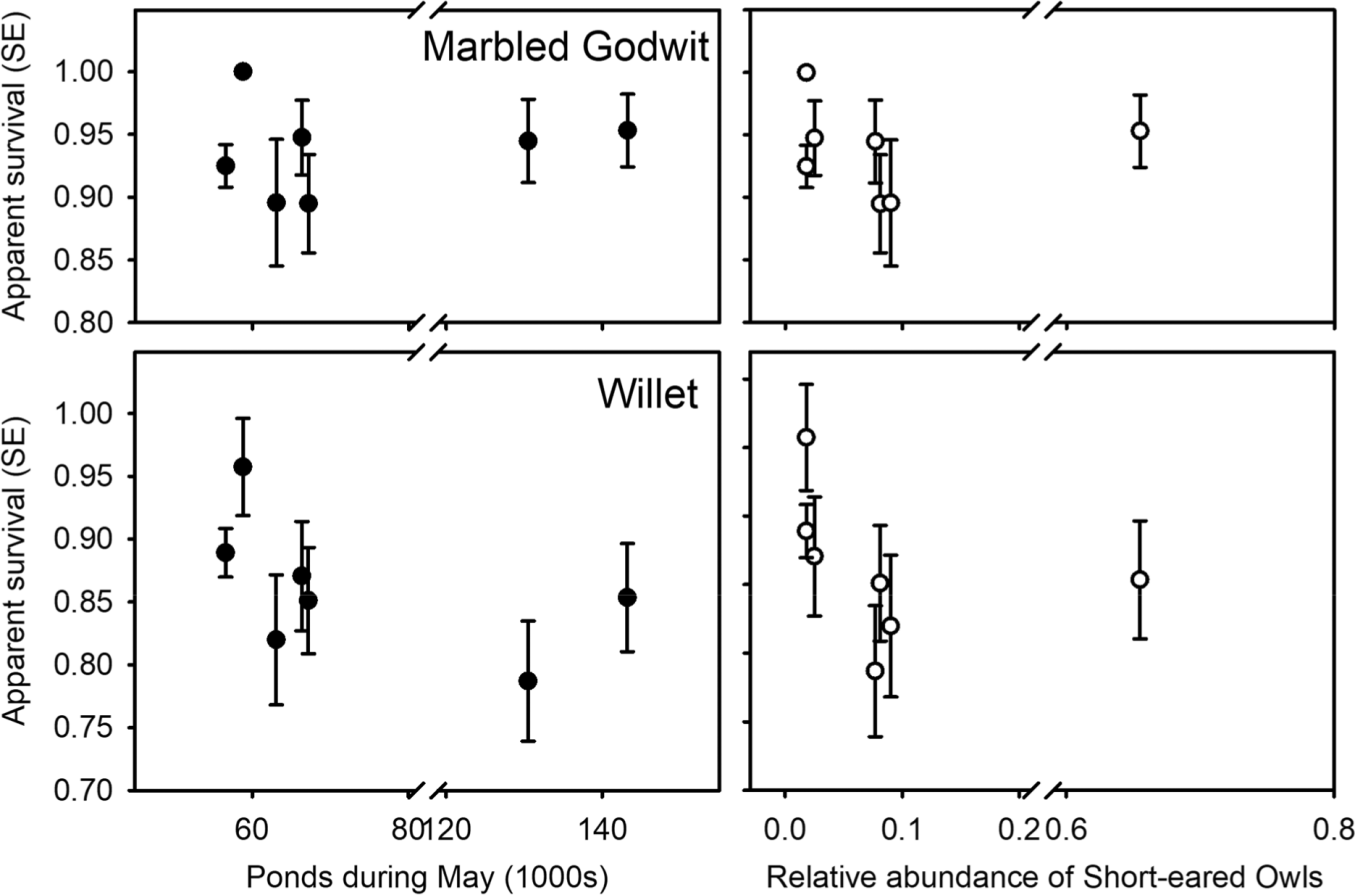
Estimates of apparent survival as a function of number of ponds and owl abundance for Marbled Godwits and Willets breeding near Brooks, Alberta, 1995–2001. Breaks in the *x*‐axis were used for clarity due to large differences among years in the two explanatory factors. Parameter estimates for apparent survival taken from models *ϕ*(year), *p*(con).

Models where apparent survival was modeled as a function of annual variation in water levels or relative abundance of Short‐eared Owls were parsimonious in both species of shorebirds (Table 3). Parameter estimates from an unconstrained model, *ϕ*(year), *p*(con), indicated that apparent survival tended to be higher in two wet years (1996–1997) for godwits (Figure 2, top left) but was lower among willets (Figure 2, bottom left). However, coefficients for the effects of water conditions were not significantly different from zero in either godwits (*β* = +0.40, 95%CI = −0.65 to +1.46) or willets (*β* = −0.34, 95%CI = −0.90 to +0.24). Moreover, analysis of deviance showed that annual variation in water conditions did not explain significant variation in apparent survival for either godwits (9.7%, *F*_1,4_ = 0.43, *p* = .55) or willets (20.3%, *F*_1,4_ = 1.02, *p* = .37). Estimates of apparent survival from a factorial model with effects of water levels and sex, *ϕ*(sex × ponds), *p*(con), showed that water levels had a greater effect on females than males (Table 4). In years of high water levels, the apparent survival of female godwits was 4 points higher, whereas the apparent survival of female willets was 5 points lower. The relative abundance of owls had poor performance as an explanatory factor compared to water levels. Apparent survival of godwits and willets in the year with high numbers of rodents and high relative abundance of owls (1997) was not unusual compared to the other study years (Figure 2, right panels). Coefficients for the effects of owl abundance were not significantly different from zero in either godwits (*β* = +0.45, 95%CI = −0.99 to +1.90) or willets (*β* = 0.005, 95%CI = −0.77 to +0.78). Owl abundance did not explain annual variation in apparent survival for either godwits (7.1%, *F*_1,4_ = 0.31, *p* = .61) or willets (0.1%, *F*_1,4_ < 0.001, *p* > .99).

**TABLE 4:** Estimates of apparent survival ($\hat{\phi }$) and probability of encounter ($\hat{p}$) for Marbled Godwits and Willets at the Kitsim wetland complex near Brooks, Alberta, 1995–2001.

Sex	Year	Parameter	$\hat{\theta }$	SE ($\hat{\theta }$)	95% CrI ($\hat{\theta }$)
*Marbled Godwits*					
Females	Wet	$\hat{\phi }$	0.9479	0.0290	0.8780, 0.9888
	Dry	$\hat{\phi }$	0.9114	0.0279	0.8506, 0.9587
Males	Wet	$\hat{\phi }$	0.9521	0.0264	0.8882, 0.9893
	Dry	$\hat{\phi }$	0.9382	0.0234	0.8862, 0.9765
		$\hat{p}$	0.9161	0.0176	0.8785, 0.9474
Females	All years	$\hat{\phi }$	0.9244	0.0212	0.8782, 0.9610
Males		$\hat{\phi }$	0.9438	0.0184	0.9032, 0.9751
		$\hat{p}$	0.9154	0.0176	0.8781, 0.9466
Both sexes	All years	$\hat{\phi }$	0.9337	0.0146	0.9029, 0.9596
		$\hat{p}$	0.9157	0.0174	0.8787, 0.9470
*Willets*					
Females	Wet	$\hat{\phi }$	0.7863	0.0448	0.6923, 0.8674
	Dry	$\hat{\phi }$	0.8403	0.0331	0.7713, 0.9012
Males	Wet	$\hat{\phi }$	0.8652	0.0381	0.7821, 0.9303
	Dry	$\hat{\phi }$	0.8887	0.0272	0.8309, 0.9368
		$\hat{p}$	0.8812	0.0194	0.8405, 0.9167
Females	All years	$\hat{\phi }$	0.8164	0.0266	0.7636, 0.8676
Males		$\hat{\phi }$	0.8819	0.0224	0.8319, 0.9186
		$\hat{p}$	0.8866	0.0189	0.8449, 0.9191
Both sexes	All years	$\hat{\phi }$	0.8494	0.0176	0.8120, 0.8815
		$\hat{p}$	0.8865	0.0190	0.8449, 0.9191

*Note*: Parameter estimates were taken from MCMC sampling of models *ϕ*(sex × ponds), *p*(con); *ϕ*(sex), *p*(con); and *ϕ*(con), *p*(con).

Despite marking and resighting relatively large samples of birds over a 7‐year study period, the minimum AICc models for both species had a relatively simple structure with either effects of sex or a constant probability of apparent survival (Table 3). Parameter estimates based on MCMC sampling showed that overall apparent survival was high in Marbled Godwits ($\hat{\phi }$ = 0.934 ± 0.015SE) and tended to be higher among males ($\hat{\phi }$ = 0.944 ± 0.018SE) than females ($\hat{\phi }$ = 0.924 ± 0.021SE, Table 4). We applied the method of moments approach to decompose variance components among the parameter estimates from *ϕ*(year), *p*(con), and estimated apparent survival with process variance alone to be $\hat{\phi }$ = 0.953 ± 0.012SE. In willets, models with an effect of sex received more than twice the support of a constant model (ratio of AICc weights: 0.229/0.103 = 2.2×), and apparent survival was again higher among males ($\hat{\phi }$ = 0.882 ± 0.022SE) than females ($\hat{\phi }$ = 0.816 ± 0.027SE, Table 4). Overall apparent survival from model phi(con), *p*(con) was also relatively high at $\hat{\phi }$ = 0.849 ± 0.018SE and if estimated with the variance components procedure was $\hat{\phi }$ = 0.861 ± 0.015SE. Based on our annual estimates of apparent survival, the expected life expectancy ($\hat{E}$) of birds first captured as adults at the breeding grounds would be 14.6 years for Marbled Godwits and 6.1 years for willets (Table 5). Small sexual differences in either true survival or site fidelity had a large effect on life expectancy because the apparent survival rates were close to the boundary of one. Thus, predicted life expectancy would be 12.7 and 17.3 years for female and male godwits and 5.0 and 7.7 years for female and male willets.

**TABLE 5:** Predicted years of life expectancy ($\hat{E}$) for Marbled Godwits and Willets first captured as adults at the Kitsim wetland complex near Brooks, Alberta, 1995–2001.

Species	Sex	$\hat{E}$	SE ($\hat{E}$)	95% CrI ($\hat{E}$)
Marbled Godwits	Male	17.3	5.8	9.8, 39.7
	Female	12.7	3.7	7.7, 25.1
	Both sexes	14.6	3.3	9.8, 24.3
Willets	Male	7.7	1.5	5.4, 11.8
	Female	5.0	0.8	3.7, 7.0
	Both sexes	6.1	0.8	4.8, 7.9

### 3.4 Mate and site fidelity

**FIGURE 3:**
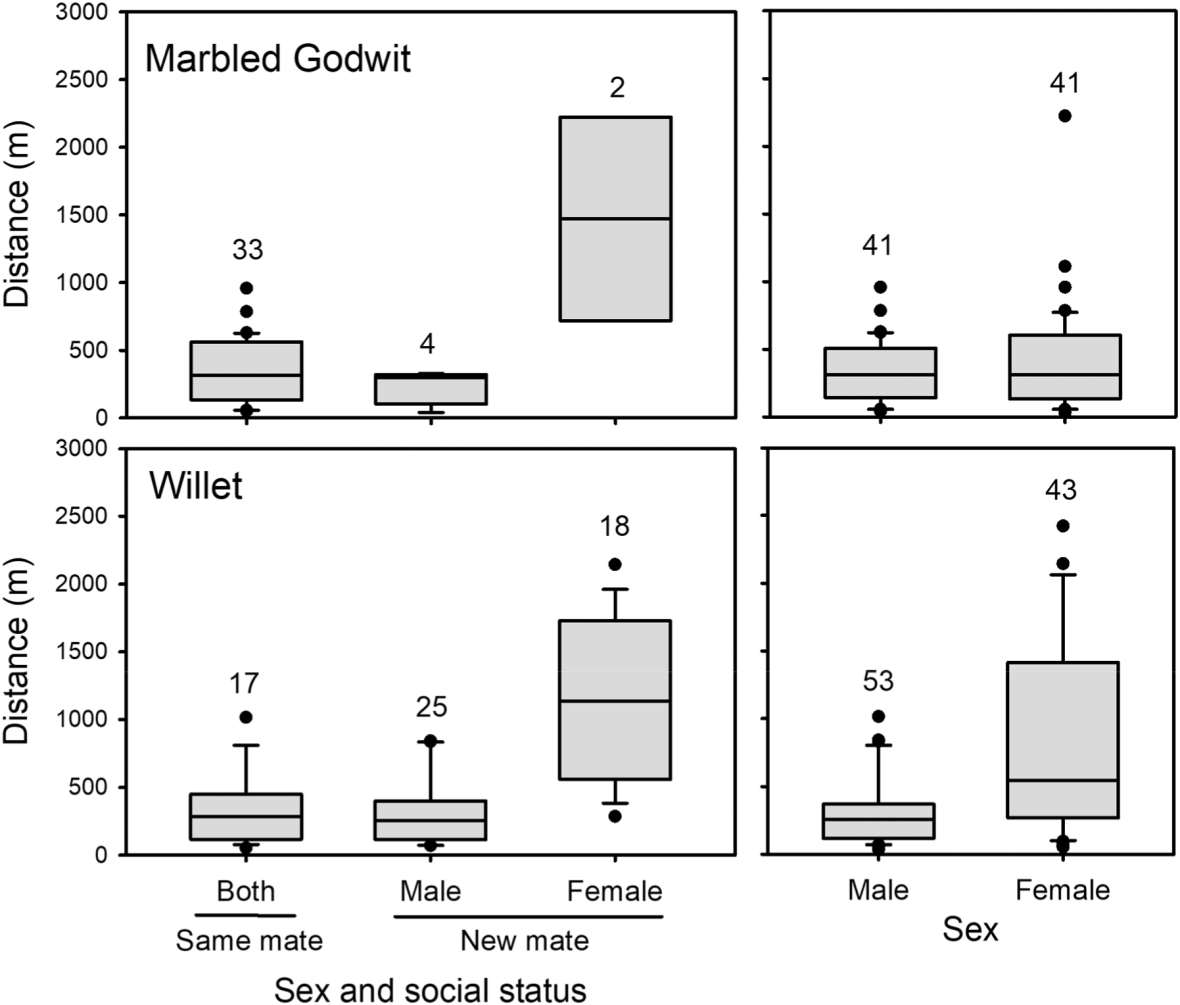
Estimates of breeding dispersal between years by social status and sex for Marbled Godwits and Willets breeding near Brooks, Alberta, 1995–2001. Box plots indicate median, interquartile range, 95%CI, and outliers, with sample sizes at top.

Adult Marbled Godwits and Willets showed strong site fidelity if returning to breeding territories at the Kitsim wetland complex in different years (Figure 3). Marbled Godwits had high rates of mate fidelity and most pairs reunited (85%, 33 of 39 pairs) with only a few cases where a bird was found to have changed mates (15%, 6 of 33, Figure 3, top left). Distances among nests in different years were similar for pairs that reunited (median = 315 m, range = 51–958 m, *n* = 33) and males with a new mate (301 m, 41–327 m, *n* = 4), but were significantly longer for two females that changed to a new mate (1472 m, 720–2223 m, one‐way ANOVA: *F*_2,36_ = 14.6, *p* < .001). Willets had lower rates of mate fidelity and pairs that reunited (28%, 17 of 60) were less common than birds changing to a new mate (72%, 43 of 60, Figure 3, bottom left). Nevertheless, patterns of breeding dispersal in Willets were similar to Marbled Godwits. Distances among nests in different years were again similar for pairs that reunited (median = 283 m, range = 52–1016 m, *n* = 18) and males with a new mate (255 m, 69–841 m, *n* = 25), but were longer for females known to have changed mates (1138 m, 286–2143 m, *F*_2,57_ = 31.2, *p* < .001). We pooled birds of known and unknown mating status to compare breeding dispersal between the sexes. Breeding dispersal distances were not significantly different between female (median = 315 m, range = 31–2223, *n* = 41) and male godwits (315 m, 41–958, *n* = 41, two‐sample *t*‐test with unequal variances: *t*_63.8_ = 0.81, *p* = .42, Figure 3, top right). However, male willets showed stronger site fidelity to Kitsim and had shorter breeding dispersal distances (median = 255 m, range = 35–106 m, *n* = 53) compared to females (547 m, 52–6010, *n* = 43, *t*_44.9_ = 3.66, *p* < .001, Figure 3, bottom right). Similar patterns were also found for within‐year breeding site fidelity if a pair lost their first clutch but laid a renest. All female Marbled Godwits that renested remained with the same mate and moved short distances (median = 315 m, range = 120–587 m, *n* = 4). Female Willets that renested and retained the same (*n* = 5) or an unknown mate (*n* = 2) moved short distances (median = 323 m, range = 158–457 m), whereas one female that changed partners moved 1443 m to lay a renest.

## 4 DISCUSSION

### 4.1 Apparent survival of adults

Our overall estimates of apparent survival for breeding populations of Marbled Godwits ($\hat{\phi }$ = 0.953) and Willets ($\hat{\phi }$ = 0.861) at the Kitsim wetland complex in southern Alberta are among the highest estimates of annual survival reported for any breeding or nonbreeding population of large‐bodied species of godwits (*Limosa* spp.), curlews (*Numenius* and *Bartramia* spp.), or shanks and tattlers (*Tringa* spp., Table 6). Among godwits, only a meadow‐breeding population of black‐tailed godwits (*Limosa limosa*) in the Netherlands have been reported to have a similar rate of annual survival ($\hat{\phi }$ = 0.95, Schroeder et al., 2010). Our high estimates of apparent survival for Willets were comparable to rates reported for nonbreeding populations of Common Redshanks (*Tringa totanus*) in Wales, UK ($\hat{\phi }$ = 0.846, Burton et al., 2006) and Green Sandpipers in England (RR = 0.835, Smith et al., 1992). Our annual estimates of apparent survival indicate that projected lifespan could be 17.3 years for male godwits and 7.7 years for male willets. Estimates of maximum longevity are known to be biased because they are a function of ringing and recovery effort (Sandercock, 2003). Nevertheless, our estimates of average lifespan are consistent with previous reports of maximum longevity for both species, including 25–29+ years for godwits (Colwell et al., 1995; Gratto‐Trevor, 2020) and 10+ years for willets (Lowther et al., 2020). Large‐bodied shorebirds are expected to be long lived because longevity records of 15–25+ years have been reported for several other species of curlews and shanks (Gill et al., 2010; Klima et al., 2013; Marks, 1992; Minton et al., 2011; Sandercock & Kramos, 2020).

**TABLE 6:** Annual estimates of return rates (RR), apparent survival (*ϕ*), and true survival (*S*) from population studies of four genera of large‐bodied shorebirds (*Limosa*, *Numenius*, *Bartramia*, and *Tringa*).

Species	Study site	Season	Method	Estimate	Source
Godwits (*Limosa*, 4 of 4 spp) and Curlews (*Numenius*, 4 of 8 spp, and *Bartramia*, 1 of 1 sp.)					
Marbled Godwit *Limosa fedoa*	Alberta, Canada	Breeding	*ϕ*	0.953	This study
	California, USA	Nonbreeding	RR	0.350	Kelly and Cogswell (1979)
Hudsonian Godwit *Limosa haemastica*	Alaska, USA	Breeding	*ϕ*	0.740	Swift et al. (2020)
	Chile	Nonbreeding	*ϕ*	0.821	
Bar‐tailed Godwit *Limosa lapponica*	New Zealand	Nonbreeding	*ϕ*	0.910	Conklin et al. (2016)
	England, UK	Breeding	RR	0.880	Evans and Pienkowski (1984)
	Australia	Nonbreeding	*ϕ*	0.71–0.93	Piersma et al. (2016)
Black‐tailed Godwit *Limosa limosa*	Netherlands	Breeding	*ϕ*	0.950	Schroeder et al. (2010)
	England, UK	Nonbreeding	*ϕ*	0.87–0.94	Gill et al. (2001)
	Netherlands	Breeding	RR	0.810	Groen and Hemerik (2002)
	Netherlands	Breeding	*ϕ*	0.830	Roodbergen et al. (2008)
Long‐billed Curlew *Numenius americanus*	Idaho, USA	Breeding	RR	0.850	Redmond and Jenni (1986)
Eurasian Curlew *Numenius arquata*	England, UK	Breeding	*ϕ*	0.920	Robinson et al. (2020)
	Wales, UK	Nonbreeding	*ϕ*	0.899	Taylor and Dodd (2013)
	United Kingdom	Breeding	*S*	0.898	Cook et al. (2021)
	Sweden	Breeding	RR	0.858	Berg (1994)
Whimbrel *Numenius phaeopus*	Shetlands, UK	Breeding	RR	0.890	Grant (1991)
	Chile	Nonbreeding	*ϕ*	0.860	Andres et al. (2018)
	Manitoba, Canada	Breeding	RR	0.649	Johnson et al. (2016)
	Iceland	Breeding	RR	0.644	Katrínardóttir et al. (2015)
	Manitoba, Canada	Breeding	RR	0.621	Skeel (1983)
Bristle‐thighed Curlew *Numenius tahitiensis*	Hawaii, USA	Nonbreeding	*ϕ*	0.910	Ruthrauff et al. (2019)
	Hawaii, USA	Nonbreeding	*ϕ*	0.852	Marks and Redmond (1996)
Upland Sandpiper *Bartramia longicauda*	Kansas, USA	Breeding	RR	0.381	Mong and Sandercock (2007)
Shanks and tattlers (*Tringa*, 6 of 13 spp)					
Willet *Tringa semipalmata*	Alberta, Canada	Breeding	*ϕ*	**0.861**	**This study**
	Virginia, USA	Nonbreeding	RR	0.731	Howe (1982)
	California, USA	Nonbreeding	RR	0.650	Kelly and Cogswell (1979)
	California, USA	Breeding	RR	0.520	Haig et al. (2002)
Lesser Yellowlegs *Tringa flavipes*	Alaska, USA	Breeding	RR	0.670	Tibbitts and Moskoff (2020)
Wood Sandpiper *Tringa glareola*	Poland	Migration	*ϕ*	0.710	Minias et al. (2010)
Wandering Tattler *Tringa incana*	Alaska, USA	Breeding	RR	0.720	Gill et al. (2020)
Common Greenshank *Tringa nebularia*	Scotland, UK	Breeding	*ϕ*	0.633	Nethersole‐Thompson and Nethersole‐Thompson (1979)
Green Sandpiper *Tringa ochropus*	England, UK	Nonbreeding	RR	0.835	Smith et al. (1992)
Common Redshank *Tringa totanus*	Wales, UK	Nonbreeding	*ϕ*	0.846	Burton et al. (2006)
	Sweden	Breeding	*ϕ*	0.797	Ottvall (2005)
	Scotland, UK	Nonbreeding	*ϕ*	0.740	Insley et al. (1997)
	England, UK	Breeding	*ϕ*	0.736	Thompson and Hale (1993)
	Ukraine	Breeding	RR	0.724	Zhmud (1992)

Despite substantial variation in water levels from year to year and some differences in rodent numbers and predator abundance, we found little evidence for annual variation or effects of annual covariates on apparent survival of godwits or willets. Our ability to detect effects of environmental covariates was relatively low in a 7‐year project. Nevertheless, a lack of annual variation is consistent with the overall high estimates of apparent survival that we found for both species in our study. Our work is consistent with previous population studies of shorebirds in showing that adult survival is often buffered against year‐to‐year variation in environmental conditions in long‐lived species (Ottvall & Härdling, 2005; Weiser et al., 2018). Species with a slow life‐history strategy can be vulnerable to environmental change if anthropogenic factors cause additive mortality (Sandercock et al., 2011; Watts et al., 2015), but they may be less susceptible to environmental stochasticity if demographic buffering is important (Hilde et al., 2020).

### 4.2 Breeding site fidelity and mate fidelity

We found small sexual differences in apparent survival with males having higher apparent survival than females in both species. Higher return rates or apparent survival among males are a common feature of shorebirds that are socially monogamous with a male‐territorial social system (Kwon et al., 2022; Oring & Lank, 1984; Sandercock & Gratto‐Trevor, 1997; Thompson & Hale, 1993), although no sex differences have been reported too (Groen & Hemerik, 2002; Ottvall, 2005). Mated pairs of shorebirds usually winter at separate nonbreeding sites but meet again at breeding sites (Gratto‐Trevor, 2011; Gunnarsson et al., 2004). Our estimates of breeding dispersal indicated that males returned to the same territory, but females move farther than males when changing mates, similar to patterns previously reported in some *Calidris* sandpipers (Gratto et al., 1985; Sandercock et al., 2000; van Leeuwen & Jamieson, 2018). Similarly, sex differences have been reported in homing rates to the same breeding territory in Eurasian Curlews (*Numenius arquata*, male 91% vs. females 78%, Berg, 1994) and Common Redshanks (*Tringa totanus*, males 84% vs. females 67%, Jackson, 1994) and to the same breeding population in Whimbrels (*N. phaeopus*, males 87% vs. females 68%, Grant, 1991). Our core study area was 21.6 km^2^ in area and dispersal movements >2 km would have led to permanent emigration from most of the area. The longest dispersal movements detected in our study were 1–6 km. Thus, sex differences in apparent survival can be due to differences in true survival, but here might also be explained by sex differences in breeding site fidelity.

Marbled Godwits and Willets had high annual survival and similar patterns of breeding site fidelity, but unexpectedly, their rates of mate fidelity were quite different. Most pairs of godwits retained the same mate (85%, 33 of 39 pairs) whereas less than a third of willets reunited (28%, 17 of 60 pairs). Rates of mate fidelity are variable in other socially monogamous shorebirds, with estimates ranging from 46% in Common Redshanks (Hale & Ashcroft, 1982), 67% in Western Sandpipers (*Calidris mauri*, Sandercock et al., 2000), 80%–94% in Semipalmated Sandpipers (*C. pusilla*, Gratto et al., 1985; Sandercock et al., 2000), 89% in Black Turnstones (*Arenaria melanocephala*, Handel & Gill, 2000), 92% in Pacific Dunlin (*C. alpina*, van Leeuwen & Jamieson, 2018), and up to 95% for Eastern Willets breeding in a saltmarsh habitat (Howe, 1982). A range of hypotheses have been proposed to account for interspecific variation in mate‐fidelity rates, including explanations that relate mate fidelity to habitat preferences, reproductive success, mortality rates, or aspects of migration (Choudhury, 1995; van Leeuwen & Jamieson, 2018). Mate fidelity is often lower among shorebirds that breed in ephemeral habitats (Friedrich et al., 2015), but seems unlikely to explain species differences here because godwits and willets bred sympatrically in the same managed wetlands. Similarly, variation in reproductive success cannot explain species differences in mate fidelity because nest success was similar for godwits (54%, *n* = 114 nests) and willets (47%, *n* = 202 nests, authors personal observation). Higher rates of mate fidelity were consistent with high annual survival and the longer life expectancy of Marbled Godwits. In other long‐lived birds with a socially monogamous mating system such as Black Brant (*Branta bernicla*), breeding with a familiar mate can improve breeding propensity and subsequent survival (Leach et al., 2020). Last, both species have wide nonbreeding ranges but Marbled Godwits are short‐distance migrants that winter in southern California and Mexico (Gratto‐Trevor, 2020; Warnock et al., 2021), whereas Willets migrate long distances to winter in South America (Martínez‐Curci et al., 2014; Oswald et al., 2016). Pairs of Willets may reunite less frequently if they are more likely to arrive asynchronously at the breeding grounds after northbound migration.

## 5 CONCLUSIONS

Our findings of high annual survival rates of Marbled Godwits and Willets set a new ceiling on the demographic performance of breeding shorebirds and provide an important baseline for evaluating new methods and the effects of future environmental change. New tracking technologies can provide valuable insights into the movements of migratory shorebirds, but it is often difficult to assess whether the methods are also reducing survivorship (Olson et al., 2014; Senner et al., 2019). Estimates of survival for banded‐only birds provide a useful comparison for evaluation of different types of harness designs and tracking tags (Hill et al., 2019; Mong & Sandercock, 2007; Ruthrauff et al., 2019; Watts et al., 2019). Baseline estimates of survival are also useful for identifying the negative effects of habitat loss and degradation. For example, Piersma et al. (2016) found that annual survival of Bar‐tailed Godwits (*Limosa lapponica*) dropped from 0.89–0.93 to 0.71–0.80 following loss of major stopover sites in the east Asian flyway. Apparent survival of a wintering population of Common Redshanks dropped from 0.85 to 0.78 following impoundment of an intertidal mudflat with a barrage for flood control (Burton et al., 2006), and apparent survival of wintering Eurasian Curlews dropped from 0.95 to 0.81 during 2 years with mechanized dredging of cockles in a coastal estuary (Taylor & Dodd, 2013). Reductions in apparent survival may be explained by increased mortality rates or by greater displacement from local areas. Estimates of vital rates can also provide insights into the demographic mechanisms that underly population dynamics. For example, high adult survival rates indicate that low reproductive rates are the likely driver of population declines among Eurasian Curlews and Black‐tailed Godwits in western Europe (Franks et al., 2017; Robinson et al., 2020; Roodbergen et al., 2012).

High annual survival rates and strong site fidelity demonstrate that managed wetlands provide an important breeding habitat for godwits and willets in Alberta. Conservation easements for waterfowl production may not benefit all wildlife species (Koper & Schmiegelow, 2006), but did create suitable breeding conditions for prairie shorebirds (Gratto‐Trevor, 2006). Additional effort to improve habitat conditions are unlikely to improve adult survival but could create breeding opportunities for more birds. Recoveries of dead birds in our study area indicated that demographic losses can occur during the breeding season with predation and powerline collisions as the two most important causes of mortality. Predation and collision mortalities are also important sources of mortality for other grassland birds, including prairie chickens (Wolfe et al., 2007), Upland Sandpipers (*Bartramia longicauda*, Mong & Sandercock, 2007), and Sandhill Cranes (*Grus canadensis*, Murphy et al., 2016). In the past, powerlines may have been routed through prairie wetlands because lands in agricultural production had greater value. Future measures to reduce collision mortalities could include precautions to avoid siting of new infrastructure near wetland habitats where shorebirds may be vulnerable during aerial courtship displays, or by marking of powerlines and wind turbines to increase visibility (Barrientos et al., 2011). In other populations of migratory shorebirds, legal and illegal harvest are also major sources of mortality (Andres et al., 2022; Redmond & Jenni, 1986; Watts et al., 2019). Measures to prevent unsustainable harvest require identification of important harvest zones and implementation of improved regulations (McDuffie et al., 2022). For example, annual survival of Eurasian Curlews increased after a hunting ban from ca. 0.72 to 0.85 at two wintering sites in southern England (Cook et al., 2021). We conclude that reducing exposure to anthropogenic mortality will be important for conservation of large‐bodied shorebirds because low natural mortality rates imply that any additional losses are likely to lead to additive mortality (Sandercock et al., 2011; Watts et al., 2015).

## AUTHOR CONTRIBUTIONS

**Brett K. Sandercock:** Conceptualization (equal); data curation (equal); formal analysis (lead); writing – original draft (lead); writing – review and editing (equal). **Cheri L. Gratto‐Trevor:** Conceptualization (equal); data curation (equal); formal analysis (supporting); funding acquisition (lead); investigation (lead); methodology (lead); project administration (lead); writing – original draft (supporting); writing – review and editing (equal).

## FUNDING INFORMATION

Canadian Wildlife Service (Environment Canada, Prairie and Northern Region); Alberta North American Waterfowl Management Plan Centre (Biodiversity Fund).

## Data Availability

Datasets from the project and R scripts for the analyses are publicly available at the Dryad archive (https://doi.org/10.5061/dryad.3n5tb2rmq).
